# Therapeutic Use of MicroRNAs in Lung Cancer

**DOI:** 10.1155/2014/756975

**Published:** 2014-09-16

**Authors:** Orazio Fortunato, Mattia Boeri, Carla Verri, Massimo Moro, Gabriella Sozzi

**Affiliations:** Tumor Genomics Unit, Department of Experimental Oncology and Molecular Medicine, Fondazione IRCCS Istituto Nazionale dei Tumori, Via Venezian 1, 20133 Milan, Italy

## Abstract

Lung cancer is a leading cause of cancer deaths worldwide. Although the molecular pathways of lung cancer have been partly known, the high mortality rate is not markedly changed. MicroRNAs (miRNAs) are small noncoding RNAs that actively modulate cell physiological processes as apoptosis, cell-cycle control, cell proliferation, DNA repair, and metabolism. Several studies demonstrated that miRNAs are involved in the pathogenesis of lung diseases including lung cancer and they negatively regulate gene and protein expression by acting as oncogenes or tumor suppressors. In this review we summarize the current knowledge on the role of miRNAs and their target genes in lung tumorigenesis and evaluate their potential use as therapeutic agents in lung cancer. In particular, we describe methodological approaches such as inhibition of oncogenic miRNAs or replacement of tumor suppressor miRNAs, both in *in vitro* and *in vivo* assays. Furthermore we discuss new strategies to achieve *in vivo* tissue specific delivery, potential off-target effects, and safety of miRNAs systemic delivery.

## 1. Introduction

Lung cancer is the most frequent cause of cancer mortality worldwide, accounting for more than 1.4 million deaths per year [1]. The two major histotypes of lung cancer are nonsmall cell lung cancer (NSCLC) (about 80% of all lung cancers) and small cell lung cancer (about 20%) [2].

Despite improvements in early diagnosis and new therapeutic strategies, the overall 5-year survival remains only of 10–20% [1]. The poor prognosis is due to diagnosis at advanced stage, tumor heterogeneity, and relatively limited understanding of lung cancer biology. Surgical resection is so far the most common treatment for early stages tumors, in combination with chemotherapeutic agents for advanced lung cancer patients or chemotherapy treatment alone for metastatic disease. The platinum-based treatment is commonly used in the clinical practice, with small benefit in survival of lung cancer patients. The identification of driver mutations and genetic rearrangements in approximately 50–60% of NSCLC has led to a change in the treatment of lung cancer patients [3–5], by identifying subgroups of patients characterized by different molecular profile. K-RAS mutations have been found in approximately 17% of all NSCLC, especially in adenocarcinomas (27%–34%), whereas the discovery of activating mutations in the EGFR gene (23%) and rearrangements of anaplastic lymphoma (ALK) (5%) were found and had also relevant impact in the treatment of lung cancer patient, through their responsiveness to tyrosine kinase inhibitor (TKI) agents, such as erlotinib, crizotinib, and gefitinib [6, 7].

Although new targeted therapies entering clinical trials have shown positive results, a large number of patients still remain without any potential known target for therapy. Therefore, the identification of novel treatment strategies is critical and essential for lung cancer management.

MicroRNAs (miRNAs) are small noncoding, 22 nt-long, RNAs that are able to bind complementary sequences of target mRNAs and to induce either their degradation or translational repression [8]. In mammals, miRNAs control the activity of more than 50% of all protein-coding genes [9]. MiRNAs are expressed in a tissue or developmental specific manner, thereby greatly contributing to cell-type-specific profiles of protein expression. MiRNAs potentially target hundreds of different mRNAs and thus coordinate or fine-tune the expression levels of many proteins, thus regulating a wide variety of cellular processes [10]. To date, more than 1000 human miRNAs were found in the genome and a considerable number of them were found deregulated in cancer cells compared to normal cells [11–14].

The rationale of miRNA therapy in lung cancer is based on two criteria: one is that miRNAs play an essential role in lung development [15] and their expression levels are deregulated in lung cancer compared to normal tissues [16] and also in the blood of patients [17] and the second one is that several studies demonstrated that modulation of miRNA expression, both* in vitro* and* in vivo*, can modify the cancer phenotype [18–22].

Different strategies of miRNAs therapeutics can be envisaged according to the expression status of miRNAs in the tumor: (i) miRNAs inhibition when it is overexpressed using antagomir, which are synthetic miRNAs complementary to the miRNAs of interest or miRNAs sponges, that compete with the targets of the miRNA and (ii) miRNAs replacement using oligonucleotide mimics or viral vectors when the miRNAs is downmodulated [23].

In this review, the role of those miRNAs, which regulate fundamental proteins and pathways involved in lung tumorigenesis, will be discussed. Furthermore, we will describe general therapeutic strategies involving inhibition of oncogenic miRNAs and replacement of tumor suppressor miRNAs including recent experimental methods developed to bypass blood clearance and poor intracellular uptake.

## 2. miRNA Role in Lung Carcinogenesis

Human cancers exhibit an altered expression of miRNAs with oncogenic or tumor-suppressive activity providing an explanation of their role in tumor development and progression. Recent studies reported an aberrant expression of miRNAs in lung tumor tissues when compared to the corresponding normal lung tissues, supporting miRNAs involvement in lung carcinogenesis (Table 1). Lethal-7 (let-7) is one of the earliest identified tumor suppressor miRNA in lung cancer and its downmodulation is associated with poor prognosis [16, 24–27]. Furthermore, let-7 can regulate several oncogenic pathways; for example, this miRNA negatively modulates multiple cell-cycle oncogenes as KRAS, mutation of which are commonly implicated in lung adenocarcinomas [28] MYC and HMGA2 [29, 30]. Furthermore, an involment of let-7 in the regulation of miRNA maturation process-Dicer mediated has also been described [31]. Let-7 overexpression in lung adenocarcinoma A549 cell line inhibits* in vitro* assays cell growth and reduces cell-cycle progression by targeting CDC25A, CDK6, and cyclin D2 [32]. Accordingly,* in vivo* experiments show that let-7b inhibits lung cancer cell xenografts in immunodeficient mice [33]. In addition, the therapeutic potential of let-7 in lung cancer treatment is supported also by experimental evidence that altered expression of let-7 family members associated with radiation resistance [34].

Mir-34 is a tumor suppressor miRNA largely investigated in cancers and its deregulation has been reported in various tumor types, including lung cancer [35–39]. Mir-34 members (a, b, and c) are regulated by p53 and mir-34 expression is frequently reduced in p53 mutant tumors [40]. mir-34 family members are involved in cell-cycle control, apoptosis, and cellular senescence through specific targeting of BCL-2, MYC, and MET genes [41]. In addition, Garofalo et al. showed that mir-34a and mir-34c are downregulated in NSCLC cell lines and described an inverse correlation between PDGFR-*α*/*β* and miR-34a/c expression in lung tumor samples [42]. The inhibition of PDGFR-*α* and PDGFR-*β* by miR-34a/c replacement antagonizes tumorigenicity and increases sensitivity to TRAIL-induced cell death, suggesting an important therapeutic application for lung cancer. The potential therapeutic use of mir-34 is further supported by Kasinski and Slack, who investigated the effects of miR-34 replacement on tumor formation and progression in a mouse model [43].

Unlike let-7 and mir-34, mir-21 has been described as a oncogenic miRNA, and it was found overexpressed in lung cancer [44]. The expression levels of mir-21 were significantly higher in tissues from NSCLC patients with lymph node metastasis than in patients without lymph node invasion [45] and the diagnostic and prognostic values as biomarkers were confirmed in several works [45–49]. These data suggest that the overexpression of mir-21 plays a critical role in lung tumorigenesis.* In vitro* inhibition of mir-21 expression in NSCLC cell lines showed a deregulation of cellular mechanisms as programmed cell death, cellular proliferation, and migration. Validated target for mir-21 include TPM1, PDCD4, and PTEN [50–53] and recently mir-21 overexpression was indeed associated with the acquired resistance of EGFR-TKI in NSCLC, due to the activation of PI3K/AKT pathway through PTEN and PDCD4 inhibition by mir-21 [54]. The role of mir-21 as an oncogenic miRNA and its therapeutic value were also demonstrated in an* in vivo *assay using K-ras^LA2^ murine model of multifocal lung cancer, where the downmodulation of mir-21 expression resulted in a reduction of tumor number, incidence, and size [55].

Oncogenic activity was also reported for mir-17/92a cluster (mir-17, mir-18a, mir-19a, mir-20a, mir-19b, and mir-92), that is, upregulated in several cancer types, both hematopoietic and solid tumors [56–59]. miRNAs from this cluster are regulated directly by c-MYC and have a central role in the control of cellular proliferation [60]. Reporter assays revealed target sequences for mir-19a and mir-19b-1 in the 3′UTR of PTEN [61] and the introduction of mir-19a and mir-19b-1, or the full cluster, was sufficient to induce c-MYC-driven B-cell lymphoma development in mouse model by restoring PTEN expression levels [57, 62]. In lung cancer, this cluster is frequently amplified and in particular mir-17-5p and mir-20a are overexpressed. These miRNAs are fundamental for lung cancer development by targeting HIF-1a, PTEN, BCL2L11, CDKNA, and TSP-1 [63].

Several papers showed that mir-15a/16 are implicated in cell-cycle control and likely contribute to NSCLC tumorigenesis [64–67]. mir-15a/16 are frequently deleted or downregulated in squamous cell carcinomas and adenocarcinomas of the lung, and their expression is inversely correlated with the expression of cyclin D1. In NSCLC cell lines, physiologic concentrations of mir-15a/16 regulated cyclin D2 and cyclin E1 expression. Using luciferase reporter constructs of the different cyclin genes, miRNAs were able to downregulate luciferase activity and this effect was reverted by cotransfection with anti-mir-15a/16. In addition, mir-15a/16-induced cell-cycle arrest can be partially restored by overexpression of CCND1 or CCNE1.

Recent works investigated mir-200 family (mir-200b, mir-200a, mir-429, and mir-200c) and its role in the promotion of EMT in NSCLC through regulation of ZEB1 transcription factors and regulation of CDH-1 and vimentin expression [68–70]. In addition, in a mouse model that develops metastatic lung adenocarcinoma, overexpression of mir-200b locus in highly metastatic cells eliminated their ability to undergo EMT and metastasize and also this miRNA could be used to distinguish lung tumors cell lines based on their site of origin, with higher levels in cells from a primary tumor than in cell lines derived from metastatic sites. Recently Tejero and colleagues demonstrated that high levels of miR-141 and miR-200c are associated with shorter overall survival in a cohort of NSCLC patients with adenocarcinoma [71–73].

Among the reported miRNAs that are downregulated in lung cancer, the miRNA-29 family (29a, 29b, and 29c) expression in lung cancer tissue is associated with DNA methyl-transferase DNMT-3A and DNMT-3B levels, two important enzymes for DNA methylation that are frequently upregulated and associated with poor prognosis [74]. The expression of mir-29 family members in lung cancer cell lines restored normal patterns of DNA methylation, inducing reexpression of tumor suppressor genes such as FHIT and WWOX that were previously silenced by methylation, thereby inhibiting tumorigenesis* in vitro* and* in vivo*. These findings support the role of mir-29s in epigenetic normalization of nonsmall cell lung cancer (NSCLC), providing a rationale for the development of miRNA-based therapeutic interventions for the treatment of lung cancer.

Finally, mir-221 and mir-222 are reported to be involved in lung cancer by targeting PTEN and TIMP3 tumor suppressors, inducing TRAIL resistance and enhancing cellular migration [75, 76]. Mir-221/222 are modulated by both epidermal growth factor (EGF) and MET receptors and were found to be involved in gefitinib resistance in cooperation with mir-30b and mir-30c, by inhibiting APAF1 and BIM cell death genes. Since MET amplification plays an important role in the resistance to anti-EGFR agents, a modulation of these miRNAs could have therapeutic applications to sensibilize lung tumors to TKI therapy [77].

Recently, the work of Hu et al. identified a new member of the miR-548 family in the intron of human FHIT gene, which is a tumor suppressor gene altered in human cancer as lung, head and neck, esophageal, stomach, pancreatic, and cervical cancer. This miRNA inhibited human tumor cell growth* in vitro* and* in vivo* xenograft mouse model targeting CCND1, ERBB2, DNMT3A, and DNMT3B [78].

## 3. miRNA as Therapeutic Agents

Delivery of miRNAs as synthetic miRNA mimics or antagomirs has emerged as a promising approach to treat cancer (Table 2). To date, MRX34 is the only phase I clinical trial for miRNA replacement in patients with primary liver cancer or with liver metastasis from other cancers (Mirna Therapeutics). MRX34 is based on the formulation of miR-34 mimic and the liposomal delivery technology SMARTICLES (Marina Biotech). Interestingly, mir-34, as mentioned above, is one of the most deregulated tumor suppressor miRNA in lung cancer suggesting also a potential use of this methodology for the treatments of lung cancer patients.

The first evidence of miRNAs replacement in lung cancer was reported in 2008. The authors showed that restoring let-7 expression affected tumor growth in xenograft models derived from lung cancer cells H460 or A549 (NSCLC cell lines carrying mutations in K-RAS gene) injected subcutaneously in immunodeficient mice. In this work, cells were transiently transfected with 30 nM of let-7 miRNA mimic prior to subcutaneous injection into NOD/SCID mice and a delay in tumor growth was observed. They also used a conditionally inducible K-RAS mutated mouse model (Kras^LSL-G12D/+^) developing orthotopic lung adenocarcinoma to demonstrate that let-7 expression interferes with lung tumorigenesis. They treated mice intranasally with adenovirus expressing cre recombinase (Ad-cre) (5 × 10^8^ pfu) to induce lung cancer growth and treatment with a second adenovirus expressing let-7 resulted in significant tumor growth inhibition [33].

Trang and coworkers investigated the therapeutic role of let-7 in established lung tumors [79]. They injected intratumorally 6.25 *μ*g of let-7 mimics mixed with siPORT-amine (siPORT), a lipid-based reagent, in xenograft mouse models of H460 lung cancer cell line. Mice were treated on days 11, 14, 17, and 20 after xenograft implantation and tumor size measure on day 21 resulted in a robust tumor growth inhibition. This group also obtained a reduction in tumor growth with intranasal injections of lentiviral (10^6^ pfu) or adenoviral (5 × 10^8^ pfu) vectors expressing let-7 in Kras^LSL-G12D/+^ mice. Local delivery by intratumorally or intranasal injection of miRNA demonstrated a significant tumor reduction but is probably inadequate for the clinical setting of miRNA therapy.

In 2010, Wiggins et al. reported a tumor growth reduction by miR-34 treatment in H460 and A549 NSCLC xenograft models in immunodeficient mice [80]. NOD/SCID mice were treated with different concentration of mir-34 mimic (5 and 1 mg/kg) formulated with MaxSuppressor* in vivo* RNALancerII, a neutral lipid emulsion (NLE) delivery reagent (BIOO Scientific, Inc.), injected intratumorally or intravenously on days 12, 15, and 18 after xenograft implantation. Interestingly, they performed a blood chemistry analysis, to test possible toxicity in liver, kidney, and heart, and observed that miRNA treatment was well tolerated. Using a different mouse model they proved that miRNA treatment did not induce nonspecific immune responses in mice by measuring serum cytokines. In summary, this study provided evidence for the safe and effective therapeutic delivery of a miRNA mimics. However, further studies have to demonstrate that miRNAs replacement has no effect on normal cells.

One year later, the same authors also showed that using NLE, rather than the standard cationic liposomes, it was possible to transfect miRNAs systemically by intravenous injections in mice and these particles with miRNA were taken up especially by lung tissue, thus showing that NLE could vehicle therapeutic miRNAs to lung tumors. Indeed, using NLE, Kras^LSL-G12D/+^ mice were treated with 1 mg/kg of let-7 or mir-34 for 8 consecutive days and showed a significant reduction of tumor burden [81]. The authors suggest that NLE may bypass some of the shortcomings that can be attributed to charge of cationic lipids. These particles form less aggregates in biofluids, are not filtered by the liver, and do not adhere to the endothelium or are taken up by macrophages [82].

More recently, it was created a double transgenic mouse model, Kras^LSL-G12D/+^; Trp53^LSL-R172H/+^, where KRAS mutation was induced by Ad-cre in the cells with a p53 null background to recapitulate human lung carcinogenesis [43]. In this study, mir-34 was able to reduce progression of preformed tumors and also totally prevented tumor formation. For these experiments mice were intubated and 3 × 10^6^ TUs of lentiviral particles expressing mir-34 were administrated directly in the thoracic region.

New methodologies for miRNA delivery were settled up in order to reduce cytotoxicity, to avoid liver capture, and to better target lung cells. Among these, the biodegradable cationic polyurethane short branch polyethylenimine (PU-PEI) system was conjugated with mir-145 and showed very high transfection efficiency when injected intratumoral in lung adenocarcinoma cancer stem cells (LAC-CSCs) xenograft models, suppressing the stem-like properties and sensitizing them to chemo- or radiotherapy [83]. These nonviral gene vectors could represent a genetically and immunogenically safe delivery method for miRNAs-based therapies, but since intratumoral administration is not a feasible clinical route, PU-PEI should be subjected to further characterization for systemic delivery.

In an effort to find an appropriate delivery method of miRNAs for lung cancer treatment, Shi et al. used solid lipid nanoparticles (SLNs) containing dimethyldioctadecylammonium bromide (DDAB) that condense miRNAs and enhance cellular uptake. They demonstrated that intravenously injection of 0.5 mg of SLNs loaded with Cy3-miR-34 was taken up by lung tissue in just 1-2 hours, resulting in a cytosolic delivery of the miRNA [84]. Their results indicate that miSLNs enhance miR-34a uptake into the lung, due to the ability of the delivery vehicle to avoid liver capture and to be physically entrapped in the capillary of the lung compared to lipofectamine particles, which were captured by liver. Recently, Wu et al. developed a cationic lipoplexes-based carrier system containing miR-29b (LP-miR-29b) to treat mice by intravenous injection and reducing tumorigenicity of the A549 xenograft models [85]. LPs formulation contained DOTMA, a cationic lipid that confers a positive surface charge for cellular uptake, cholesterol to improve transfection efficiency and protect from degradation, and a short polyethyleneglycol molecule linked to vitamin E to increase the stability and circulation time of LPs* in vivo*. Furthermore, Cortez et al. developed a systemic delivery method using miR-200c-loaded liposomes (NOV340/miR-200c; SMARTICLES) to enhance radiosensitivity in a mouse model of lung cancer. They described mir-200c accumulation in tumor, liver, brain, and lung after subcutaneous injection in mice. Interestingly, NOV340/miR-200c is a liposomal nanoparticle that contains a synthetic, double-stranded mimic of the tumor suppressor miRNA miR-200c which is composed of amphoteric lipids that change their net charge depending on the pH of the environment, such that it becomes cationic at low pH and anionic at higher pH, to prevent unwanted interactions and to adhere only at tumor site [86].

## 4. Challenges for miRNA Therapy

The principal advantage of using miRNA as therapeutics agents is that they could target several genes of redundant pathways involved in cancer development [87]. For this reason, a small number of miRNAs could be used to achieve a broad silencing of protumoral pathways and they become more attractive than mixture of small interfering RNAs already used for therapy. In addition, miRNA mutations are extremely rare due to the small size of the sequence and probably a potential resistance to miRNA therapeutics would require multiple mutations in several genes. Furthermore, several studies demonstrated that relatively small changes in miRNAs and their corresponding targets expression could induce phenotypic alterations, reinforcing the idea that correction of a limited number of miRNA could revert the malignant phenotype [88]. The challenges for the use of microRNA in therapy, that have to be addressed, are tissue specific delivery, potential off-target effects and safety.

In this review, we described several strategies of miRNAs replacement/inhibition such as intratumoral injections and regulation of miRNAs expression by viral vectors which represent delivery routes unlikely to be applied in a clinical context. Intratumoral injections could be used only for a small number of easily accessible tumors. Similarly, modulation of miRNAs expression with viral vectors could potentially show the same weaknesses encountered in gene therapy, such as limited infectivity and problems in the transcription of the gene product. In addition, cancer cells frequently show deficiencies in the maturation of miRNA precursors so expression with a viral vector is a less preferable approach.

Thus, the most promising approach for miRNAs therapeutics is the systemic delivery of miRNA mimics or inhibitors. The main obstacle for the systemic delivery of miRNAs in lung cancer therapy is to determine their uptake in lung cancer cells; this delivery has to overcome miRNAs degradation by nucleases, renal clearance, failure to cross the capillary endothelium, ineffective endocytosis by target cells, and activation of the host immune responses [89–92]. A major aspect to be considered is how to deliver the therapeutic molecules into the target cells without inducing unwanted responses in cells other than the intended ones. Furthermore, other potential side effect of miRNAs therapy is that the same miRNA can affect hundreds of targets genes in different cell types and regulates different mechanisms. Therefore, miRNA modulation might have beneficial effects in one cell type but harmful effects in another. Further complication is the finding that miRNAs generated from each strand of the same hairpin RNA structure, termed 5p and 3p, may bind to different mRNAs and display different behaviour. For example, it has been reported that miR-125a-5p and miR-125a-3p, which are downregulated in NSCLC, exhibit distinct effects* in vitro* on the migration and invasion of lung cancer cells [93]. This information has direct implications for the design of miRNA gene therapy; in fact, when using a precursor miRNA inserted into a viral vector and both strands are produced, the function of each strand in the cells must be identified.

## 5. Conclusion

The challenges for the future development of miRNA therapy as the improvement of stability, delivery, and control of off-target effects of miRNAs need to be addressed.

We hope that some of the miRNAs strategies described above will be further developed to improve specificity and efficacy and finally will be used, alone or in combination with chemotherapy, for the treatment of lung cancer patients.

## Figures and Tables

**Table 1:** Principal miRNAs involved in lung carcinogenesis.

miRNAs	Expression in lung cancer	Cellular processes affected and targets	Reference
Let-7 family	Decreased	(i) Cell proliferation (KRAS, MYC, and HMGA2)	[28–30]
		(ii) miRNA maturation Dicer mediated	[31]
		(iii) Cell-cycle regulation (CDC25A, CDK6, and cyclin D2)	[32]

mir-34 family	Decreased	(i) Transcriptionally activated by p53	[40]
		(ii) TRAIL-induced cell death and cell proliferation (BCL-2, MET, and PDGFR-α/*β*)	[41–43]

mir-21	Increased	(i) Apoptosis, cellular proliferation, and migration (TPM1, PDCD4, and PTEN)	[50–53]
		(ii) TKI-treatment resistance	[54]

mir-17/92a cluster	Increased	(i) Transcriptionally regulated by c-MYC	[60]
		(ii) Cellular proliferation and cancer development (PTEN, HIF-1a CL2L11, CDKNA, and TSP-1)	[57, 61–63]

mir-15a/16 cluster	Decreased	Cell cycle regulation (cyclin D1, D2 and E1)	[66, 67]

mir-200 family	Decreased	Promotion of EMT and metastasization (ZEB transcription factors,CDH-1, and vimentin)	[68–73]

miRNA-29 family	Decreased	Epigenetic regulation of gene expression (DNMT-3A and DNMT-3B)	[74]

mir-221/mir-222	Increased	(i) TRAIL resistance and cellular migration (PTEN and TIMP3)	[75, 76]
		(ii) Transcriptionally regulated by EGFR and MET and gefitinib resistance (BIM and APAF1)	[77]

mir-548	Decreased	Tumor cell growth (CCND, ERBB2, DMNT3A, and DNMT3B)	[78]

**Table 2:** Strategies for *in vivo* miRNA delivery.

	Type of particles	Advantages	Disadvantages	Reference
Synthetic	Mimic inhibitor	Safe and simple Easy to produce Low immunogenicity	Poor cellular uptake Rapid degradation and clearance	[94]

Neutral lipoplexes/liposomes	MaxSuppressor SLNs SMARTICLES siPORT	Low immunogenicity Lung accumulation Easy to produce Available commercially	Nonspecific uptake Low transfection efficiency Cytotoxicity	[79, 80, 84, 86]

Cationic lipoplexes/liposomes	PU-PEI DOTMA	Low immunogenicity Easy to produce High cellular uptake	Cytotoxicity Tend to form aggregates Moderate transfection efficiency Nonbiodegradability	[81, 83]

Viral vectors	Adenovirus *Lentivirus *	High transfection efficiency Stable expression	Difficult to produce High immunogenicity High toxicity	[33, 43]
